# Exploring Physiotherapeutic Approaches in the Management of Iliac Blade Fractures

**DOI:** 10.7759/cureus.69312

**Published:** 2024-09-13

**Authors:** Simran F Sheikh, Swapna Jawade, Samruddhi Aherrao, Anushka Rohankar, Ishwari Gawande

**Affiliations:** 1 Department of Musculoskeletal Physiotherapy, Ravi Nair Physiotherapy College, Datta Meghe Institute of Higher Education and Research, Wardha, IND

**Keywords:** iliac blade, numerical pain rating scale, pelvic instability, physical therapy, wound management

## Abstract

One of the leading causes of pelvic fractures, especially iliac blade fractures, is road traffic accidents (RTAs). The orientation of the iliac blade fracture and the associated pelvic instability can provide particular challenges for diagnosis, treatment, and rehabilitation. We hereby, specify a case report of a 17-year-old male suffering from several injuries, including a compound iliac blade fracture, in a high-speed motor vehicle incident. The initial assessment revealed that the pelvic area was found to be painful, swollen, and had limited mobility. Diagnostic imaging such as X-ray evaluation was used to confirm the iliac blade's compound 3B fracture. Conservative procedures comprised of immobilization, medical treatment, and physical therapy. Early mobilization, pain management, and physical therapy to regain pelvic stability and function were all part of the follow-up therapy. The cause of iliac blade fracture in the patient is RTA. The present case report outlines the standardized protocols implemented to improve outcomes associated with iliac blade fractures secondary to RTAs. It emphasizes the significance of effective rehabilitation in the early management of symptoms such as pain and decreased range of motion (ROM), as well as in preventing deformity.

## Introduction

Pelvic fractures, which have a mortality rate of 3% to 8%, are among the most serious and potentially fatal orthopedic injuries. Typically, pelvic injuries are caused by blunt traumatic injuries with large energy disruptions, such as those are falls from heights, bicycle and pedestrian collisions, and motor vehicle crashes. Several medical conditions have been associated with pelvic pain, including peri-pelvic soft tissue injuries, stomach injuries, and fractures of the extremities [1].

Compound fractures are defined in several ways. A compound fracture occurs when there is a direct connection between the fracture site and the surrounding air through a wound in the skin or connective tissue. Fractures may result in soft tissue lacerations or lacerations associated with bone fragments. Fractures can become compound over time due to poor handling, jagged bone ends piercing the skin, or breakdown of the soft tissues surrounding the fracture [2]. However, one of the most prominent pelvic structures is the iliac blade, which is also referred to as the iliac crest or iliac wing. Several muscles, ligaments, and tendons of the abdomen and back attach to the iliac blade, which forms the curving upper border of the ilium. An iliac blade fracture is a type of fracture that can result from several kinds of traumatic incidents, including car crashes, falls from a height, or direct impacts on the pelvis. Significant trauma is frequently linked to iliac blade fractures, which may also involve injuries to other areas of the pelvis, hip, or adjacent structures. A stable iliac blade or crest fracture is usually painful. Pain in the hip or groin is typical and can be exacerbated by moving the hip or attempting to walk; however, walking may still be doable. Other symptoms include pain and soreness in the groin, hips, lower back, buttocks, and pelvis, as well as bruising and swelling around the pelvic bones.

A sensation of tingling or numbness in the genitals or upper thighs. Pain may also be present while sitting or working. Additionally, there could be evident symptoms of bleeding. Bruising or sensitive lumps are present in the groin or perineum. Women are likely to suffer from vaginal bleeding, while men will experience scrotal bruises [3].

Radiographs (X-rays) are used to diagnose pelvic fractures, and they are a rapid and easy screening that detects the majority of fractures. They can be difficult to assess due to the multifaceted shape of the sacrum, pelvis, and proximal femoral. Computed tomography (CT) is the preferred imaging modality for accurately displaying complicated acetabular and pelvic ring fractures. Following an initial plain X-ray, a CT is frequently required to provide an accurate assessment of the fracture and to aid in surgical decision management [4]. The management of an iliac fracture mainly depends on the severity of the injury and the patient's health. Conservative methods of treatment for compound or stable fractures include rest, medical treatment and rehabilitation, and if required, minor surgery [5].

Treatment options include immobilization with a brace or casting and surgical fixation accompanied by physical therapy, depending on the severity of the fracture and related injuries. Physiotherapists can employ either active or passive methods to achieve their objectives. Active treatment, including exercise, requires patients to take an active role in their rehabilitation. Passive interventions, such as joint mobilization, involve the patient taking a passive role throughout the procedure [6]. Rehabilitation of an individual with an iliac fracture may be necessary to address typical symptoms, such as discomfort in the pelvic region that worsens with movement or weight-bearing activities, as well as restoring normal walking patterns and gait. In addition to assisting patients in performing these kinds of movements, the hospital's rehabilitation department actively stretches and mobilizes their joints. Whenever it comes to increasing the range of motion (ROM), continuous passive movement is among the most often used techniques.

Furthermore, strengthening exercises significantly increase the strength of hip motions and restore the active ROM of the hip joint to improve lower limb function and stability [7]. Hip and pelvic discomfort and restricted ROM can result from fractures. Exercises for rehabilitation can aid in restoring mobility and increasing flexibility in the injured area. Muscle weakness in the vicinity of the fracture may result from prolonged immobilization or decreased activity. Hip abductors stabilize the hip during gait and prevent pelvic tilting on the non-stance limb. Atrophy may impact daily tasks like walking or climbing stairs [8]. Exercises designed to improve the muscles of the hips, thighs, and core are frequently included in rehabilitation programs.

An intense rehabilitation program started on the first day following injury for a compound iliac fracture has been found to be both safe and effective [9]. The purpose of post-fracture rehabilitation care is to reduce recovery time, morbidity incidence, and financial expenditures through multidisciplinary approaches. Furthermore, a rehabilitation plan is necessary to improve daily living tasks while also maintaining the body's flexibility, strength, and balance [7].

## Case presentation

Patient information

A 17-year-old male was taken to the hospital on January 23, 2024, with severe injuries after colliding with a car while driving a motorcycle. He sustained injuries, including an open lacerated cut in the left iliac area and abrasions on multiple areas of his body. The patient was examined further with an X-ray, CT scan, and pelvic ultrasonography. The individual's treatment was managed conservatively using brace immobilization, management of an open lacerated wound, analgesics for pain, and physical therapy. The patient complained of sudden, progressive, and excruciating pain, which was aggravated by lower limb movement, whether in the supine or side-lying position, and was relieved by medication. The patient's score on the numerical pain rating scale with movement of the left lower extremity was observed as 9/10, and on rest, it was 5/10.

Timeline of treatment

The timeline of treatment is mentioned in Table 1.

**Table 1:** Timeline of rehabilitation

Date	Events
23/01/24	The patient was admitted to the orthopedic department and diagnosed with an iliac blade fracture
24/01/24	Physiotherapy rehabilitation was started. Pain and lower extremity functional scale pre-intervention assessment was taken
25/01/24	The patient’s gait training was started with a walker
30/01/24	Progression of gait training was done
07/02/24	Pain and lower extremity functional scale post-intervention assessment was taken

Investigations and its findings

**Figure 1:**
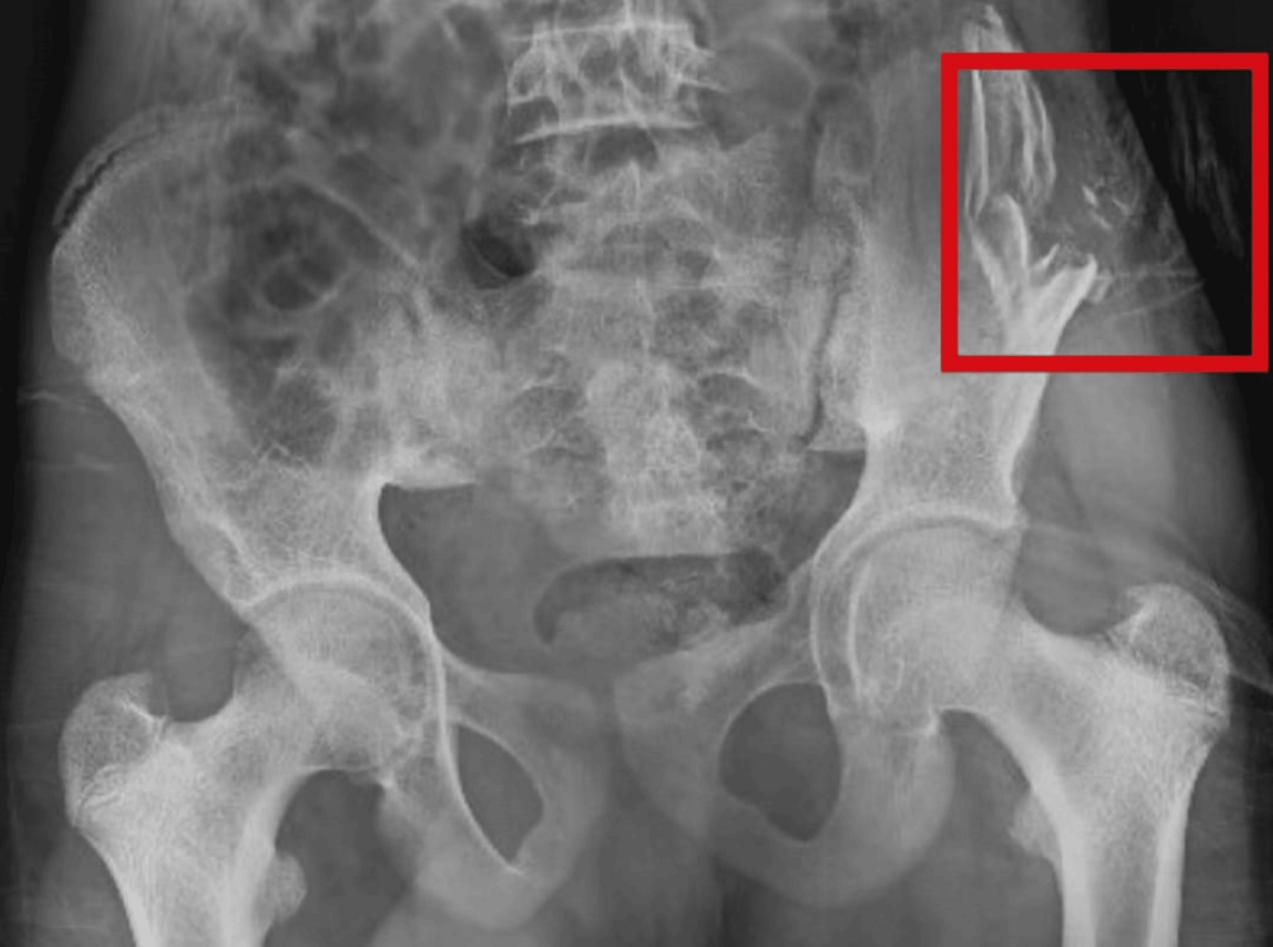
Radiographic scan, in which the red box marking shows compound grade 3B iliac blade fracture

The radiographic scan revealed a compound grade 3B fracture of the iliac blade (as mentioned in Figure 1).

Diagnosis

The patient was diagnosed with compound 3B left iliac blade or crest fracture according to the orthopedic trauma association (OTA) classification.

Clinical assessment

The patient submitted written informed consent. Throughout the general examination, the patient was conscious, cooperative, and aware of the time, location, and person. The patient was hemodynamically stable, and his vitals were normal. On observation, the patient was seen lying supine with his head elevated to 300, wearing an immobilization brace around his pelvis, and his left forearm was bandaged. The table illustrates the ROM and manual muscle testing. His numerical pain rating scale score was 9/10 on the first day of treatment. The pre-interventional assessment of a ROM is stated in Table 2, and manual muscle testing is in Table 3.

**Table 2:** Illustrating the ROM pre-intervention NA: not assessed; ROM: range of motion

Movement	Right (active)	Right (passive)	Left (active)	Left (passive)
Hip flexion	0-90°	0-100°	NA	NA
Hip extension	0-15°	0-20°	NA	NA
Hip abduction	0-25°	0-30°	NA	NA
Hip adduction	0-40°	0-50°	NA	NA
Knee flexion	0-130°	0-130°	NA	NA
Knee extension	130-0°	130-0°	NA	NA
Plantarflexion	0-45°	0-50°	0-30°	0-35°
Dorsiflexion	0-10°	0-10°	0-10°	0-10°
Inversion	0-30°	0-35°	0-25°	0-30°
Eversion	0-10°	0-10°	0-10°	0-10°

**Table 3:** Manual muscle testing pre-intervention NA: not assessed

Muscles	Right	Left
Hip flexors	3/5	NA
Hip extensors	3/5	NA
Hip abductors	3/5	NA
Knee flexors	3/5	NA
Knee extensors	3/5	NA
Ankle plantar flexors	3/5	2/5
Ankle dorsiflexors	3/5	2/5

Therapeutic intervention

Initially, the patient was managed through conservative procedures. Hence, he was conservatively managed with bed rest (immobilization), medications, and rehabilitation as he suffered from pain, a decrease in muscle strength, and decreased functional independence. The key objective of rehabilitation was to help him alleviate the pain, increase his muscular strength, promote pelvic control, and subsequently regain his functioning skills and self-reliance. Table 4 summarizes the physiotherapeutic protocol for two consecutive weeks.

**Table 4:** States about physiotherapy protocol TENS: transcutaneous electrical nerve stimulation; SLR: straight leg raise; NA: not assessed; reps: repetition

Goals	Intervention	Method	Dosage
Patient’s education	Emphasize the positive aspects of physiotherapy to the patient and his family	The effects of exercise, posture, and gait training were discussed while counseling the patient and caretakers. The pillow placement was explained to the patient and caretaker, explaining how to elevate the lower extremities with a pillow	NA
Pain management	1. Conventional TENS in pain-related dermatomes. 2. Cryotherapy	1. The frequency ranges from 50-100 Hz, with small intensity and pulse widths of 50-200μs in continuous mode. 2. Cryotherapy has been used to alleviate the pain	1. Once a day for 10-20 min, 2. two times a day for 10-12 min
Wound management	Positioning for optimal healing	1. To educate the patient to be in a semi-fowler posture and then proceed to a 90-degree upright seating, additionally, pillows beneath their abdomen and knees to help spread pressure, 2. ultrasound is helpful in the reduction of inflammation and wound healing	1. NA, 2. 7-8 min with low-frequency superior over the skin surface, given for 10 days
To increase sensory and maintain sensory function	Sensory re-education	It was done by using different textures of fabrics to stimulate sensory receptors	5 min daily
To early mobilize joint	Passive range of movement exercises	Passive straight leg raises up to the achievable range, ankle dorsiflexion, and plantarflexion	1. 10 reps*1 set, 2. After one week progression to active SLR, 3. On the seventh day, SLR with holds of 5 sec was started
To prevent stiffness	Strengthening exercises	1. Strengthening exercises for quadriceps, hamstrings, and glutes in supine. 2. Supine hamstring curls with a medicine ball were started from the seventh day	10 reps×1 set
To promote pelvic control and stability	Pelvic bridging	1. Unilateral supine pelvic bridges. 2. After one week the progression to pelvic bridges on the elevated surface was done	10 reps×1 set
To improve breathing and functional capacity	Breathing exercises	Pursed lip breathing, thoracic expansion exercises, and aerobic exercise training	5 reps×2 set, 20 min daily
Gait training	Progressive gait training	For gait training, the patient was in an erect posture with a stable trunk: 1. spot marching, 2. climbing stairs, 3. tandem walking, and 4. obstacle training encouraged	Gait training is initiated on day two for 10 min. The progression is made after a week

Follow-ups and outcomes

Following the physiotherapy rehabilitation, the patient experienced no pain or loss of ROM and was able to carry out daily activities. The patient reported improved muscle strength and ROM. The lower extremity functional scale score was 70 out of 80, and the numerical pain rating scale (NPRS) score was one out of 10. Table 5 shows the ROM, Table 6 shows manual muscle testing, and Table 7 shows outcome measures taken on the first day and the second week.

**Table 5:** Illustrating pre-intervention and post-intervention NA: not assessed; ROM: range of motion

Joint ROM	Pre-intervention	Post-intervention
Hip flexion	NA	0-100°
Hip extension	NA	0-20°
Hip abduction	NA	0-30°
Knee flexion	NA	0-130°
Knee extension	NA	130-0°
Dorsiflexion	0-30°	0-10°
Plantarflexion	0-10°	0-35°
Inversion	0-25°	0-10°
Eversion	0-10°	0-100°

**Table 6:** Illustrating manual muscle testing NA: not assessed

Muscles	Pre-intervention	Post-intervention
Hip muscles	NA	4/5
Knee muscles	NA	4/5
Ankle muscles	2/5	5/5

**Table 7:** Outcome measures taken during pre-intervention and post-intervention NPRS: numerical pain rating scale

Outcome measure scale	Day 1	Day 14
NPRS	9/10	1/10
Lower extremity functional scale	10/80	75/80

## Discussion

The following specific case study focuses on the patient's two-week physiotherapy rehabilitation following conservative management for an iliac blade fracture. Following injuries to the chest and central nervous system, pelvic fractures are the third most common cause of mortality in car crashes [10]. Iliac wing fractures are usually produced by direct high-energy trauma, such as a strike to the iliac crest [11]. Unlike other body parts, the pelvis and hip region are susceptible to high-energy injuries. One important soft tissue component of these injuries is frequently overlooked. Unrecognized soft tissue injuries surrounding the pelvis might have a negative impact on results [12]. The first steps in evaluating a pelvic trauma are to know about the mechanism of damage (especially in high-energy impact cases, which are more common in blunt trauma cases) and physical examination to rule out pelvic ring deformity or instability, pelvic or perineal hematoma or rectal urethral bleeding [3]. Conservative or non-surgical, treatment for stable fractures includes bed rest that allows healing of fractured bones, rehabilitation, and in some cases traction, which is a device used to realign broken bones, alongside pain management treatments [13].

According to a survey done by Smith, it is safe and effective for promoting early gait and balance learning. Prompt intervention is frequently employed to promote functional rehabilitation and lower the morbidity and death rates of elderly individuals suffering from hip fractures [14]. Limiting weight-bearing activities after surgery increases the patient's dependency on assistive technology and lengthens their stay in an extended care facility, which makes recovery more difficult. When patients are recovering from lower extremity fractures, osteotomies, amputations, or arthroplasties, partial weight bearing (PWB) is frequently recommended [15]. Patients continue to experience severe post-injury pain and effusion, as well as reduced exercise tolerance. The observation and early diagnosis of substitution movements, as well as their resolution through the use of analytical physiotherapy techniques and patient education, are the foundations of correctly restoring lower extremity function and functional independence in the early period [16]. The rehabilitation goals were developed, starting with mild activities and working up to full weight-bearing walking with a walker [17].

According to a recent paper, physiotherapists consider certain techniques to be essential for high-quality pelvic fracture recovery. This study suggests that integrating transcutaneous electrical nerve stimulation (TENS) with standard care for patients can lead to improved pain relief and increased walking distance. This highlights the importance of delivering high-quality care during iliac fracture rehabilitation [18]. Low-frequency currents are, therefore, the best currents for stimulating muscle contraction and facilitating sensory perceptions [19]. It is generally recognized that early, well-coordinated physical therapy rehabilitation improved functional goals over time when combined with conservative or surgical strategy. This is a major element in the successful post-operative recovery of these patients [15].

According to global guidelines, a multidisciplinary approach that provides patients with pelvic fractures with improved recovery programs must include early mobilization and physiotherapy. Helping patients regain independence in three basic mobility activities in and out of bed, sitting to stand to sit from a chair with arms, and walking with an assistive device must be the top priority for an older adult returning to their pre-fracture living arrangement without 24-hour care. Increased physical activity is also associated with these mobility goals [20].

## Conclusions

In summary, the above-mentioned case involved the implementation of active physiotherapy management in conjunction with conservative management of an iliac blade fracture, which was crucial to the patient's overall care. The multimodal strategy used by physiotherapists seeks to promote healing, manage wounds, enhance strength, relieve pain, restore movement function, and avoid consequences like stiffness and deformity related to this kind of fracture. Physiotherapy therapies are tailored to each patient's specific needs and goals by integrating functional rehabilitation approaches, exercise therapy, and pain management modalities. Early mobilization and gait training reduce the chance of subsequent issues, including joint stiffness and muscle weakness, while promoting safe and effective movement patterns.
